# Multicenter Computer-Aided Diagnosis for Lymph Nodes Using Unsupervised Domain-Adaptation Networks Based on Cross-Domain Confounding Representations

**DOI:** 10.1155/2020/3709873

**Published:** 2020-01-24

**Authors:** RuoXi Qin, Huike Zhang, LingYun Jiang, Kai Qiao, Jinjin Hai, Jian Chen, Junling Xu, Dapeng Shi, Bin Yan

**Affiliations:** ^1^PLA Strategy Support Force Information Engineering University, Zhengzhou 450001, China; ^2^Department of Radiology, Henan Provincial People's Hospital, Zhengzhou 450002, China

## Abstract

To achieve the robust high-performance computer-aided diagnosis systems for lymph nodes, CT images may be typically collected from multicenter data, which cause the isolated performance of the model based on different data source centers. The variability adaptation problem of lymph node data which is related to the problem of domain adaptation in deep learning differs from the general domain adaptation problem because of the typically larger CT image size and more complex data distributions. Therefore, domain adaptation for this problem needs to consider the shared feature representation and even the conditioning information of each domain so that the adaptation network can capture significant discriminative representations in a domain-invariant space. This paper extracts domain-invariant features based on a cross-domain confounding representation and proposes a cycle-consistency learning framework to encourage the network to preserve class-conditioning information through cross-domain image translations. Compared with the performance of different domain adaptation methods, the accurate rate of our method achieves at least 4.4% points higher under multicenter lymph node data. The pixel-level cross-domain image mapping and the semantic-level cycle consistency provided a stable confounding representation with class-conditioning information to achieve effective domain adaptation under complex feature distribution.

## 1. Introduction

Novel techniques for medical image analysis, computer-aided diagnosis algorithms, and related topics have been recently emerging. The research in these directions depends not only on the development of artificial intelligence methods but also on the promotion of big data techniques. In fact, deep learning algorithms for computer-aided diagnosis typically use realistic big medical datasets that are manually labeled. Problems pertaining to medical image analysis are complicated by the fact that clinical images of the same modality may differ if these images are generated by devices of different models, manufacturers, or imaging parameters. For big-data-driven medical diagnosis algorithms, a model trained only by one centrally acquired dataset cannot typically generalize well, a shortcoming which limits the application scope of such model [1, 2]. However, collecting data with multiple models, parameters, or locations increase the cost of manual labeling, and balancing the numbers of data samples collected from each of these factors for one dataset is complicated leading to inflexibility in practical applications. Classification of medical imaging data under such variations is usually referred to as a cross-domain classification problem. Indeed, if there is no comprehensive dataset with a balanced quantity of samples for each factor, it is difficult to achieve optimal classification performance for these factors. For example, computerized tomography (CT) imaging shows clarity variations in some tissues due to differences in equipment or parameters. If we have enough labeled data samples for one of the CT imaging devices, we can train a stable deep learning network. The CT images collected from another device may not be suitable by themselves for training a stable network if the high labeled data requirement is not met [3, 4]. In view of this, we can define the problem of unsupervised domain adaptation in deep learning as one dealing with how to use the labeled data from one domain to achieve the common recognition of two-domain images, especially when the images are unlabeled. This problem is properly handled by a multidomain robust classification algorithm, which integrates multicenter data with less manual labeling.

There are two types of methods in domain adaption: data-centric methods and subspace-centric methods [5]. On the one hand, a data-centric method finds a unified transformation that maps data from source and target domains into a domain-invariant space so as to reduce the distributional disparities of data from the source and target domains, while retaining the data attributes of the original space. The features from the domain-invariant space are used to achieve the final classification on a different domain. On the other hand, subspace-centric methods reduce domain shifts by manipulating subspaces of two domains. For example, this manipulation can be done by establishing a linear map or by using Grassmann kernels [6] so that the subspace of each domain contributes to the formation of the final map. By comparing the two types of methods, we find that the data-centric methods require extracting features into a common implicit space for classification, while the subspace-centric method transforms the target subspace to the source subspace. Utilizing the multimodal characteristics of medical data while paying attention to the flexibility considerations in practical applications, we constructed a unified network for the classification of multicenter data.

In domain adaption problems, the most commonly used methods are the adversarial training ones such as the gradient reversal layer (GRL) method or the maximum mean discrepancy (MMD) method. Ganin et al. [7] proposed computing adversarial losses in the embedded space using the gradient reversal layer (GRL). The goal of the adversarial training is to find the parameters of a domain classifier or discriminator that maximizes its classification accuracy while also setting the parameters of a generator to maximize the confusion of the domain classifier. The biggest problem with this domain classification loss is that a good domain-invariant feature should not let the discriminator know which domain it came from. Such a domain discriminator based on binary classification tends to promote the network to extract domain-specific features, which might be irrelevant to both domains. The maximum mean discrepancy (MMD) [8] is used in many studies to depict the distributional disparity between feature spaces from different domains. Tzeng et al. [9] and Rozantsev et al. [10] use MMD to measure the distance between two feature spaces and to map the source and the target spaces to a unified feature space by minimizing this distance. Furthermore, Sun and Saenko [11] use the CORAL loss to align the mean value and the variance between two domains to achieve correlation alignment. These methods, striving to characterize feature loss only, consider the distance in the feature space but do not take into account other correlative representations between the feature spaces of the source and the target domains. When the prior class-conditioning information of the source and target domains differs greatly, the similarity of the feature spaces means the loss of the class-conditioning information of the target domain, and hence higher classification errors occur in the target domain. Furthermore, domain separation networks (DSNs) [12] use four extraction feature networks to disentangle the common and domain-specific features. Through the GRL for domain similarity constraints with soft subspace orthogonality loss for difference constraints, the obtained domain-invariant features still suffer from the problems caused by the above specific losses. The domain-specific and shared features of medical images are very complicated, indeed, because of the high resolution of these images.

The unsupervised image translation problem is similar to the domain adaption problem. For a data-centric problem, whether it is an unsupervised domain-adaption problem or a style translation problem, the main purpose is to find a domain-invariant representation across the two domains. In comparison with the methods that use specific constraint losses such as the MMD or CORAL methods, a more flexible implicit-loss scheme named cross-domain image mapping is proposed for unsupervised image style translation [13, 14], by which pixel-level domain-invariant features are obtained. The unsupervised image-to-image translation (UNIT) [15] is based on a shared-latent-space hypothesis. The UNIT cross-domain reconstruction mapping, based on generative adversarial networks (GANs) such as cycle GAN [16] and coupled GAN (CoGAN) [17], forces the network to extract common implicit spatial features. Combined with a pixel-space variational autoencoder (VAE) reconstruction loss [18], the UNIT loss function uses implicit loss constraints through image reconstruction and adversarial training to achieve more flexible feature extraction [19]. Following the approach of the domain separation networks (DSNs) [12], methods that utilize diverse image-to-image translation (DRIT) [20] and multimodal unsupervised image-to-image translation (MUNIT) [21] disentangle the common and domain-specific features and then perform cross-domain reconstruction. These methods add more image reconstruction loss to the domain-invariant constraints to obtain a more realistic reconstructed image. However, such a loss function limits feature extraction and mapping. Some studies have also applied the domain-adaptation method to image translation. The XGAN method [22] uses the GRL idea instead of the pixel-level VAE loss to achieve semantic cycle consistency of the shared feature representation allowing the network to realize flexible structural transformations.

The biggest difference between the domain-adaptation problem and the image translation problem is that the former problem is characterized by a weakening of image reconstruction quality and an enhancement of the class-conditioning information in the domain-invariant space. As mentioned above, the domain-invariant space in the domain-adaptation problem should have the characteristics of being classifiable and domain invariant. The features extracted from the two domains can be reconstructed across domains, and a generated image cannot be distinguished as to which domain it originates from. The GTA [23] uses the joint generative-discriminative method to add a classification loss to the GAN loss [24] to constrain the class-conditioning information of the generated images. The pixel-level loss corresponding to two images is not used. So, the domain adaptation of the network does not depend on the fine image reconstruction. Moreover, the importance of the gradient of the classification loss for domain adaptation is verified in [23]. Because the target-domain image is unlabeled, GTA only uses the source label to back propagate the gradients. Such a method might not be well generalized for the classification in the target domain. After all, a classifier trained only with the source domain may have certain domain-specific features. To solve this problem, we use a pair of decoders to establish a cross-domain image mapping to map the labeled source domain images to the target domain. In order to realize this cross-domain mapping and make the network robust to shifts of the class-conditioning information between the source and target domains, we propose a new concept called the classification cycle consistency in the image generation process. The classification cycle consistency, which is similar to the cycle semantic loss in XGAN, can more effectively perceive changes in the class-conditioning information and provide a more flexible structural transformation while regularizing the ill-posed unpaired cross-domain mapping problem.

**Figure 1:**
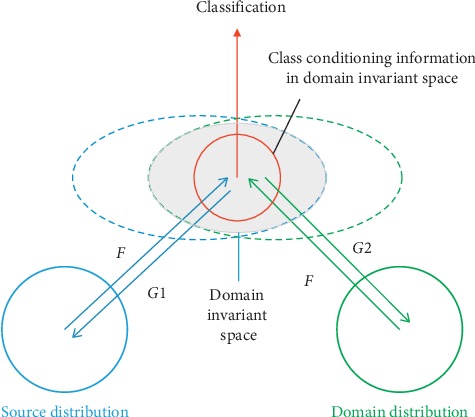
A graphical illustration of the proposed method: the cross-domain confounding representation is generated by constraining the cross-domain mapping reconstruction. The classification cycle consistency enables the network to perceive the significant discriminative representation in a domain-invariant space for final classification.

In another main contribution of this paper, a domain-adaptation network is built based on the cross-domain confounding representation to solve the multicenter problem of computer-aided diagnosis data. Inspired by the unsupervised image translation problem, a cross-domain confounding representation is used to implicitly force the network to extract domain-invariant features through cross-domain mapping. The network architecture consists of a pair of encoders and decoders, as well as a discriminator and a classifier. The pair of encoders has common parameters for feature extraction across the two domains, while the classifier achieves the final classification in the domain-invariant space. Because of the complex feature distribution of medical images and the requirement of class-conditioning information in the domain-invariant space, the classification cycle consistency is added to the loss function after the cross-domain mapping to help the network update the gradient with class-conditioning information.  Figure 1 shows a conceptual diagram of the method employed in this paper. Compared to the previous methods on domain adaptation such as those of GRL, MMD, and GTA, our method can achieve a better and more stable performance for high-resolution medical images with complex feature distributions.

## 2. Materials and Methods

We introduce here our approach in three stages. In the first stage, we introduce the network architecture and the main functions of each component. In the second stage, we combine the functions of all parts to describe the construction of the loss function. In the third stage, we introduce how the whole network uses loss function to achieve the main functions of each component and the verification of network.

### 2.1. Architecture of the Proposed Network

**Figure 2:**
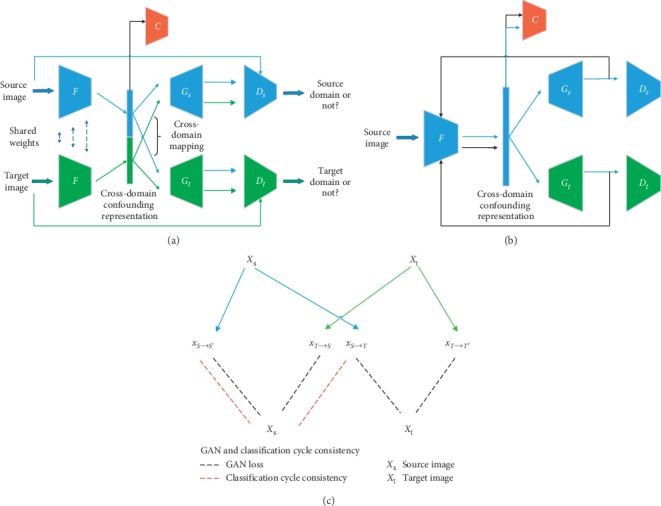
Illustration of the proposed network architecture. (a) Domain confounding representation through cross-domain mapping: the encoder F and the decoder *G* constitute the VAE architecture for unsupervised representation learning. The D module constitutes the GAN discriminator, while the C module constitutes a classifier. The encoder F uniformly encodes images from two domains. Paired decoders process different domain features, enabling cross-domain pixel-level image reconstruction and adversarial discrimination. (b) Classification cycle consistency: the reconstructed image based on source-domain features, as shown by the black line, will be constrained by classification cycle consistency through *F* and *C*. (c) Illustration of the loss overview.

Our network architecture consists of pairs of decoders and discriminators as shown in Figure 2(a). The labeled data represent the source image, and it is shown in blue while the unlabeled data represents the target image, which is shown in green. We assume that pairs of images, X1 and X2, from different domains can be mapped into a domain-invariant space. The encoder network F is a fully-weighted shared network with six convolution layers whose inputs are images from two domains. After the encoder, we apply the classification network which has two fully connected layers to classify the domain-invariant features. The decoder *G* has six deconvolution layers to reconstruct an image through the domain-invariant features. The network function structure is mainly composed of two parts. One part extracts the cross-domain confounding representation through the loss of GAN, and the other part guarantees the consistency of the class condition information in the codec process through the classification cycle consistency. Figure 2(a) indicates the process of the extraction of crossing domain confounding representation under whole network.  Figure 2(b) indicates the process of classification cycle consistency after image reconstruction through the source image phase. Features from the source domain with label information are decoded to generate an image of the corresponding domain. The generated image is then reencoded and reclassified. This update step introduces the gradient of the class-conditioning information into the decoder and encoder of the each domain and implements semantic-level consistency constraints.  Figure 2(c) shows the GAN loss and classification cycle consistency overview. Compared with the joint generative discriminative method [23], the proposed method ensures the consistency of the classification information and the domain-invariant information in the encoding and decoding processes.

### 2.2. Loss Function Construction

Let the source and target distributions be *S*(*x*, *y*) and *T*(*x*, *y*), respectively, and *S*′ and *T*′ be the generate image, respectively. Also, let the source and target images be *x*_*S*_ and *x*_*T*_. In order to achieve cross-domain confounding representation and classification cycle consistency, we optimize the domain discriminator *D*, decoder *G*, classifier *C*, and encoder *F* as follows.

#### 2.2.1. Domain Discriminator *D*

The purpose of the domain discriminator is to determine whether an input image belongs to the considered domain. The design goal is to minimize the domain discriminator loss, forcing the codec to generate images that are more in line with the domain features in the adversarial training. The application of the pixel-level adversarial losses follows the approaches of the DC-GAN [19] and the GTA [23]. To solve the problem that domain-invariant feature should not let the discriminator know which domain it came from, we apply two domain discriminators *D* in each domain so that the discriminator discriminates the domain other than the two domains. The loss function of *D* is shown in equations (1) and (2). The *S* and *T* subscripts represent the discriminators of the source and target domains, respectively:

(1) $$\begin{array}{c} {ℒ}_{D}^{S}=\underset{x_{s}∼S}{E}\underset{D_{S}}{\max}\log\left(D_{S}\left(x_{S}\right)\right)+\log\left(1-D_{S}\left(x_{S⟶S^{\prime }}\right)\right)+\log\left(1-D_{S}\left(x_{T⟶S}\right)\right), \end{array}$$

(2) $$\begin{array}{c} {ℒ}_{D}={ℒ}_{D}^{S}+{ℒ}_{D}^{T}. \end{array}$$

#### 2.2.2. Decoder *G*

The purpose of each decoder or generator *G* is to maximize the domain discriminator confusion by generating images that fool this domain discriminator as much as possible. This confusion constitutes a part of the adversarial loss. As the training progresses, the decoder *G* generates images with more domain characteristics. The classification cycle consistency (*C*(*F*(*x*_*S*⟶*S*′_)) and *C*(*F*(*x*_*S*⟶*T*_))) is introduced to update *G*. The generated images are reclassified through the encoder *F* and the discriminator *D*. By minimizing the classification loss, the decoding network captures more flexible class structure changes, retaining the class-conditioning information in the generated image. The loss function of *G* is given by equations (3) and (4). The S and *T* subscripts represent the discriminators of the source and target domains, respectively:

(3) $$\begin{array}{c} {ℒ}_{G}^{S}=\underset{x_{s}∼S}{E}\underset{G_{S}}{\min}-\log\left(C\left(F\left(x_{S⟶S^{\prime }}\right)\right)\right)+\log\left(1-D_{S}\left(x_{S⟶S^{\prime }}\right)\right), \end{array}$$

(4) $$\begin{array}{c} {ℒ}_{G}={ℒ}_{G}^{S}+{ℒ}_{G}^{T}. \end{array}$$

#### 2.2.3. Classifier *C*

The classifier *C* is applied to the domain-invariant space. The goal of the classifier is to correctly classify the source and target images, *x*_*S*_ and *x*_*T*_. For image classification, we directly optimize the classifier *C* by minimizing a binary cross-entropy loss. For the generated image, the classifier also minimizes the classification loss of the cross-domain reconstructed image in the target domain. So, the label information can be introduced in the target domain to improve the classification performance of the target-domain-specific features. It should be noted that when we use cross-domain-generated images to update *C*, we do not use the gradient information of *F* and *G*. The loss function of *C* is shown in the following equation:

(5) $$\begin{array}{c} {ℒ}_{C}=\underset{x_{t}∼\Gamma }{\underset{x_{s}∼S}{E}}\underset{C}{\min}-\log\left(C\left(F{\left(x_{S}\right)}_{y}\right)\right)-\log\left(C\left(F{\left(x_{S⟶T}\right)}_{y}\right)\right). \end{array}$$

#### 2.2.4. Encoder *F*

A part of the loss function of the encoder *F* is the same as the decoder *G*. By minimizing the loss of the classifier *C*, classification cycle consistency (*C*(*F*(*x*_*S*⟶*S*′_)) and *C*(*F*(*x*_*S*⟶*T*_))) enhances the ability to extract certain domain-specific features. The GAN adversarial loss combines this encoder loss with the domain discriminant loss. In addition, the cross-domain discriminant loss is introduced to ensure the extraction of domain-invariant features. The purpose of the generator *G* is only to generate an image of each domain to fool the corresponding domain discriminator *D* without cross-domain mapping, while the purpose of the encoder *F* is mainly to map the image to the domain-invariant space that maximizes the domain discriminator confusion during cross-domain generation. We adjust the proportion of the same-domain reconstruction loss and cross-domain reconstruction loss in *F* by the parameters *α* (0.1) and *β* (0.1). The functions of the module *F* and module *G* are different from each other in terms of the optimization process, implicitly enforcing domain invariance of the extracted features. At the same time, the goal of the encoder network *F* is to minimize the label prediction loss with L2 regularization in the cross-domain confounding representation. Equation (5) shows the loss function of the encoder *F*:

(6) $$\begin{array}{c} {ℒ}_{F}=\underset{x_{t}∼\Gamma }{\underset{x_{s}∼S}{E}}\underset{F}{\min}-\log\left(C\left(F{\left(x_{S}\right)}_{y}\right)\right)-\log\left(C\left(F{\left(x_{S⟶S^{\prime }}\right)}_{y}\right)\right)-\log\left(C\left(F{\left(x_{S⟶T}\right)}_{y}\right)\right)+\log\left(1-D_{T}\left(x_{S⟶T}\right)\right)+\log\left(1-D_{S}\left(x_{T⟶S}\right)\right)+\alpha \;\log\left(1-D_{S}\left(x_{S⟶S^{\prime }}\right)\right)+\beta \;\log\left(1-D_{T}\left(x_{T⟶T^{\prime }}\right)\right)+L_{2}\left(x_{gS}\right)+L_{2}\left(x_{gT}\right). \end{array}$$

### 2.3. Model Training

Because each module has a different loss function, the training of our network model follows a different loss update rule for each module to achieve the isolation of parameter updates. With a training input of unpaired source and target images, we alternately update the *D*, *G*, *C*, and *F* modules as mentioned in GTA [23]. For updating the discriminator *D*, we fix the parameters of all other modules and minimize the discriminator loss by moving in the direction of the gradient information of the discriminator. For the update of the decoder *G*, the parameters of the other modules are fixed, while the *G* parameters are updated by minimizing the classification cycle consistency loss and maximizing the discriminant loss. For the update of the classifier *C*, we fix all parameters except for those of the classifier and use only the source-domain images and the cross-domain generated images with the label information to minimize the classification loss under the gradient information of the encoder *F* and the classifier *C*. The objective for the encoder *F* is to maximize the domain discriminant loss, minimize the classifier loss, minimize the classification cycle consistency loss, and maximize the discriminant loss of the cross-domain generation. From the composition of the loss function, we can see that *G*, *C*, and *F* have the same part in the loss function but also have their own unique parts, so we need to update the gradient independently. On the contrary, the loss of *F* comes from *C*, *D*, and *G* at the same time. On the basis of updating *G* and *C*, it can better provide effective gradient information for *F*, thereby better optimizing the parameters of *F*. In the update order of *F*, *G*, and *C*, our principle is to use the gradient of the previous network to better train the subsequent network based on the order of the network architecture and the composition of the loss function.

### 2.4. Model Verification

**Figure 3:**
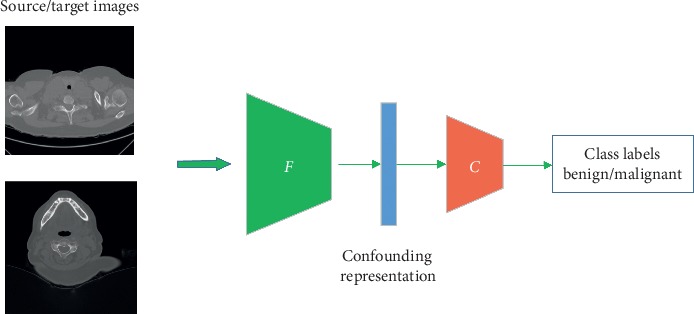
Illustration of the verification phase. *F* refers to the encoder which encodes the image to domain invariant space and *C* refers to the classifier. All those parameters are fixed during the verification.

In order to assess the domain adaptation of the proposed network, we evaluate the classification performance on a source-domain validation set and a target-domain dataset.  Figure 3 shows the verification phase, in which only the encoder and the classifier are reserved. For domain verification, the dataset is specifically divided into verification sets that are independent of the training set. Finally, the target-domain and source-domain verification accuracies are used to determine the model stability and to assess the performance of domain adaption.

### 2.5. Dataset Description

**Figure 4:**
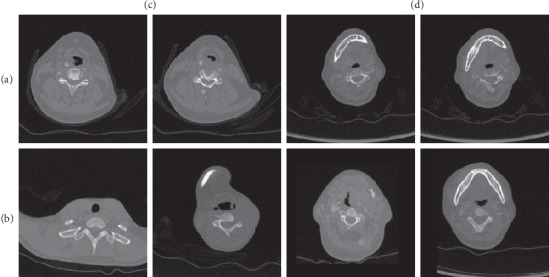
Samples of the multicenter CT images used for network training: (a) benign cases; (b) malignant cases; (c) plain CT scans; (d) enhanced CT scans.

CT images are widely used in medical examinations, and scans of different resolution are also performed in clinical diagnosis depending on the patient's condition. In particular, CT imaging has been widely used for computer-aided diagnosis of benign and malignant lymph nodes [25, 26]. During the initial diagnosis of lymph nodes, CT scans are divided into two types: plain scans and enhanced scans, where the enhanced scans show sharper details of soft tissues. A doctor will subjectively decide which scanning method to use based on the patient's initial situation. To achieve the domain adaptation for the two scan types, the trained network should eliminate well the background interference and pay attention to the class-conditioning features. The dataset used in our experiments is provided by the Department of Radiology of the Henan Provincial People's Hospital, a governmental public medical institution in China. The Department of Radiology of the Henan Provincial People's Hospital approved this study and waived the need to obtain informed consent from the patients. The enhanced CT images included 1409 malignant cases and 1099 benign cases, while the plain-scan CT images included 1310 malignant cases and 1358 benign cases. Based on the limitation of processing 2D data by our network, when processing 3D CT scan data, we extract the center slice of the lesion area as a sample from the vertical axis direction of each 3D data. The prediction of 3D images based on 2D slices can be used as the direction of later research.  Figure 4 shows benign and malignant images of the two scan types which are the two domains we address in this work. The original size of each image is 512 × 512. In order to reduce the memory consumption, we uniformly scale the images to a size of 256 × 256 before network training.

In order to verify the general applicability of our model, we also performed experiments on datasets with simple data distributions but high domain shifts. In particular, we selected the street view house number (SVHN) dataset [27] and MNIST [28] as experimental datasets. The SVHN dataset is a nonhandwritten digit dataset of color images with few structural changes, but with certain background interference. The MNIST dataset is a handwritten digit dataset of binarized images, with clear digital structure variations. To achieve domain adaptation for the two datasets, the network needs to eliminate well the background interference and accurately capture the common feature representation of the handwritten and nonhandwritten digits. We also experimented with digital datasets on a classification network of an encoder F and a classifier C for comparison with later experiments.

### 2.6. Implementation Details

Because the target-domain labels are not available in practical domain-adaptation problems, the final domain-adaptation model is generally obtained as the end-of-training model. Indeed, the model stability during training is crucial for reaching a highly optimal model in practical applications. We mainly evaluate the model performance in terms of stability and accuracy. For experimental setup, batch normalization was employed prior to the rectified linear-unit (ReLU) activation function in network training. Moreover, the Adam optimizer with a momentum of 0.99 was adopted. This framework was executed using PyTorch under the NVIDIA TITAN V 12-GB GPU.

## 3. Results and Discussion

### 3.1. Source-Only Verification Experiment

We demonstrate experimentally the classification performance based on our data and assess the subsequent domain adaptation effects on the associated source domain. In particular, we use the CT data of each single center to learn the parameters of the network architecture. The results are derived for the validation set and are shown in Table 1. Indeed, our data can achieve a good classification performance under the same deep learning model. The classification performance is similar for the cases of the two single-center CT datasets. Nevertheless, the features of the enhanced CT scans and the MNIST dataset are easier to extract and lead to a higher verification accuracy.

### 3.2. Domain Adaptation Experiments

We report here the experimental validation results of our approach. We use the commonly used domain-adaptation methods for comparison. All the mentioned procedures are reproduced in the same deep learning framework according to the reference papers. In addition to the medical dataset, we also validate our method on the simple data distributions of the digit datasets to demonstrate the universality of our method.

We test the three common domain adaptation settings. The first setting, SVHN MNIST, refers to domain adaption with SVHN as the source domain and MNIST as the target domain, while the setting MNIST SVHN refers to opposite domain-adaptation process. In the same way, the two settings, plain enhanced and enhanced plain, represent the domain adaptation on our medical dataset of the plain and enhanced CT scans.  Table 2 shows the performance under different methods, datasets, and domain settings. The performance measure is the average accuracy variance of a ten-fold validation scheme.

The source-only method means that the model was trained with the source-domain dataset and directly tested on the target-domain dataset. The network architecture is the combination of the encoder *F* and discriminator *C* networks. From Table 2, we can observe that our method significantly closes the gap between the two domain classification spaces. For the simple data distribution (MNIST and SVHN) experiment, our method almost attains the best performance obtained by GTA [23] but with lower variance value, which demonstrates the robustness of model. GRL also performed well under simple distribution data (85.4± 1.7, 87.2±2.1), while MMD performed poorly (62.6±0.7, 66.1±0.8). This difference in performance reflects that the MMD, as a loss description of feature space distance, may focus network to extract feature that is not related to class information when the prior class-conditioning information of the source and target domains differs greatly. GRL improves discrimination of domain-invariant features with more flexible adversarial losses. DSN (81.3±1.4, 86.4±0.5) uses GRL to perform similarity constraints, and by explicitly modeling the individual features of each domain, it improves the constraint of domain invariant features. The suboptimal performance of these methods reflects the rationality extraction of domain invariant feature through cross-domain confounding representations based on adversarial loss.

For the complex data distribution of the CT images, our method achieves the best performance (73.8±0.9, 72.5±1.3). The worse performance of DSN (58.5±4.1, 55.2±3.4) shows the complexity between domain-invariant features and domain-specific features under complex data distributions. GRL (65.4±3.9, 60.2±2.3) and MMD (63.5±2.7, 65.7±3.2) do not perform well on complex data distributions compared with the source-only performance, which demonstrate the effectiveness of class-conditioning information on domain-invariant spaces. The other methods show large fluctuations in network performance stability when the features become complex. Meanwhile, the performance of our method is the most stable and is also robust to the shift of the class-conditioning information in the complex feature distribution. This stability makes our method more applicable in practice for domain adaptation. Compared to the results of Table 1 and the source-only method, the results of all domain-adaptation methods are close to the results of training the same classification network with images of a specific domain under supervised conditions. We can make some observations about the verification accuracy of the source and target domains. For the CT image dataset, the accuracy of the target-domain verification will rise slowly. The source-domain verification accuracy will first rise rapidly, decline, and then rise slowly. Compared to the highest source-domain accuracy, the final stable source-domain accuracy is lower than the highest initial value, but corresponds to a higher target-domain accuracy.

We run additional experiments to assess the effect of the classification cycle consistency on domain adaptation and thus verify the importance of the class-conditioning information in domain-invariant spaces. Specifically, we test the proposed framework with and without the classification cycle consistency loss. The results are shown in Table 3. Compared with models without category information constraints, the introduction of category information is critical and useful. The network with a classification cycle consistency loss has better accuracy and stability (73.8±0.9, 72.5±1.3) under complex data distribution than a network without this loss (67.6±1.4, 65.4±1.1). The results show that the classification cycle consistency, as a method of adding class-conditioning gradients in the *F* and *G*, constrains the class-conditioning feature in domain invariant space. Compared with the result from GTA (Table 2), if it was not the classification cycle consistency for the proposed method, the performance is worse. The reason for analysis is that the GTA model introduces class information to the encoder through the classification loss of discriminator. These two results show that, no matter which domain-invariant feature extraction method, the introduction of class-conditioning information is more critical and useful. We can see that our classification cycle consistency concept not only imposes consistency constraints on the cross-domain mapping but also ensures flexible perception of the class-conditioning information and leads to a higher accuracy and a more stable performance.

## 4. Discussion

To solve the computer-aided diagnosis for muticenter lymph node data, a domain adaptation network used for complex data distribution is proposed. Unlike other domain adaptation methods, the proposed unsupervised domain adaptation method extracts domain-invariant feature through cross-domain confounding representations so that it can get a domain-invariant space for target and source data. Moreover, the classification cycle consistency captures the class-conditioning information in domain-invariant space in order to solve the difficult-to-perceive-detail class-conditioning information. The effectiveness of the proposed method is proved through the experimental results. Meanwhile, the proposed method achieves the more robust performance under both simple data distribution and complex data distribution, which may be because of the available constraint of the class-conditioning feature in domain invariant space. Moreover, it is found that the cross-domain confounding representations based on adversarial training are also robust for the change of updata sequence. Although the accuracy rates are higher than 70% through domain adaptation, it is much lower than what it should be. Maybe it is because the weight for each loss constraint needs to be well selected to balance the effect of various constraints on different parts. Furthermore, the domain adaptation performance is also needed to be demonstrated under 3D image application.

## 5. Conclusions

This paper proposes a domain adaptation method to solve the multicenter problem of the computer-aided diagnosis for lymph nodes. Aiming at the high-resolution and complex feature distribution of the lymph CT images, this paper constructs a confounding representation of domain features based on a cross-domain mapping to achieve domain-invariant feature extraction. For the complex data distribution of lymph CT images, the classification cycle consistency guides the proposed model to perceive significant classifiable representations in a domain-invariant space. Through the above method, our model achieves a stable domain adaptation of high-resolution images in complex medical field. The experimental results for simple data distributions also show the versatility of the proposed method. The training stability also allows us to simply get the optimal model under the target domain, which can further achieve the multidomain medical data integration.

## Figures and Tables

**Table 1:** Accuracy values on different datasets using the verification network model.

	Plain CT scan	Enhanced CT scan	SVHN	MNIST
Verification accuracy (%)	84.6	88.4	98.4	99.2

**Table 2:** Accuracy values (mean ± std%) with different models, datasets, and domain settings.

	MN⟶SV	SV⟶MN	Enhanced⟶plain	Plain⟶enhanced
Source only	73.1±1.4	68.3±1.5	61.5±2.3	61.6±3.5
GRL [7]	85.4±1.7	87.2±2.1	65.4±3.9	60.2±2.3
MMD [8]	62.6±0.7	66.1±0.8	63.5±2.7	65.7±3.2
DSN [12]	81.3±1.4	86.4±0.5	58.5±4.1	55.2±3.4
GTA [23]	92.5±1.2	92.4±1.3	69.4±1.1	67.4±1.8
Ours	91.6±0.3	91.8±0.4	73.8±0.9	72.5±1.3

**Table 3:** Effect of the classification cycle consistency on the classification accuracy.

	Enhanced⟶plain	Plain⟶enhanced
Without classification cycle consistency	67.6±1.4	65.4±1.1
With classification cycle consistency	73.8±0.9	72.5±1.3

## Data Availability

The code used in the research can be obtained from https://pan.baidu.com/s/1-m6PckJgM9v_JI-hCUg50g.
